# SocioLex-CZ: Normative estimates for socio-semantic dimensions of meaning for 2,999 words and 1,000 images

**DOI:** 10.3758/s13428-025-02907-9

**Published:** 2025-12-08

**Authors:** Mikuláš Preininger, James Brand, Adam Kříž, Markéta Ceháková

**Affiliations:** 1 Faculty of Arts, Charles University, Prague, Czechia; 2 Department of Psychology, Edge Hill University, Ormskirk, UK

**Keywords:** Semantics, Norms, Socio-semantics, Concepts, Representation

## Abstract

When we encounter words, we activate not only the social information provided by the speaker, but also the rich semantics of the words’ meaning, and quantifying this information is a key challenge for the cognitive and behavioural sciences. Although there are many resources available that quantify affective and sensorimotor information, there are relatively few resources available that provide information on social dimensions of meaning. We present the SocioLex-CZ norms, where the primary focus is on socio-semantic dimensions of meaning. Across two experiments, we introduce normative estimates along five dimensions—gender, political alignment, location, valence and age—for a large set of Czech words (Experiment 1) and images (Experiment 2) from 1,709 participants. We provide a series of analyses demonstrating that the norms have good reliability, and present exploratory analyses examining how the variables interact with one another within and between words/images. These norms present a valuable dataset that quantifies socio-semantic representations at scale, which we hope will be used for a range of novel and multidisciplinary applications, thereby opening up new pathways for innovative research. We make the data, code and analysis available at https://osf.io/pv9md/ and also provide an interactive web app at https://tinyurl.com/sociolex-cz-app.

## Introduction

Words have rich and diverse meanings that vary along different semantic dimensions; however, not all of the dimensions contribute to the representations of related concepts to the same extent. For example, the sensory dimension of olfaction is very important for the word aroma, but is less important for amoeba (Lynott et al., 2020). Quantifying representations has been a crucial aim of cognitive science, psycholinguistics and other interdisciplinary sciences for decades, with the pioneering work of Osgood et al. (1957) popularising the practical applications of collecting datasets of subjective ratings (or norms) along definable dimensions of meaning for lists of words.

Recent interest in such datasets has been exemplified through the growing body of studies publishing rating norms for a wide range of dimensions that capture specific aspects of meaning (e.g. humour: Engelthaler & Hills, 2018; iconicity: Winter et al., 2024; survival: Alonso et al., 2021), even investigating different language settings (e.g. multiple languages: Łuniewska et al., 2016, 2019; sign languages: Sehyr et al., 2021; second languages: Ferré et al., 2022; Imbault et al., 2021). Despite this diversity in the literature, there has traditionally been a concentrated empirical focus on two clusters of semantic dimensions, specifically affective (e.g. valence, arousal and dominance: Warriner et al., 2013) and sensorimotor (e.g. body–object interaction: Pexman et al., 2019; concreteness: Brysbaert et al., 2014; perceptual and action: Lynott et al., 2020), with these norms facilitating research on how languages are learnt, processed and represented in the brain. However, there is growing interest in moving beyond psycholinguistic, affective and sensorimotor dimensions of meaning, and instead looking towards other theoretically important and empirically valuable properties of meaning (see e.g. Binder et al., 2016), to further inform models of semantic representation and open up new avenues for researchers to better understand linguistic and cognitive processes.

One emerging perspective posits that social experiences, contexts and interactions play an important role in explaining how certain concepts can be grounded in the human conceptual system, with a particular emphasis on the grounding of abstract concepts (Barsalou, 2020; Borghi et al., 2019; Pexman et al., 2023). To date, two large-scale studies have quantified the socialness of thousands of words in English (Diveica et al., 2023) and Chinese (Wang et al., 2023), whereby socialness is defined broadly to capture a range of potential features that are socially relevant, e.g. ‘*a social characteristic of a person or group of people, a social behaviour or interaction, a social role, a social space, a social institution or system, a social value or ideology, or any other socially relevant concept*’ (Diveica et al., 2023, p. 463), and has been shown to have a facilitatory effect in a range of lexical processing tasks (Diveica et al., 2024). This would suggest that social semantic information can be used as a possible source of grounding for semantic representations, particularly when sensorimotor information is less readily available, thus providing support for multiple representation theories that have long posited a role of social experience in the representation of concepts (Barsalou, 2020; Borghi et al., 2019).

Although the expansive definition provided for the socialness norms facilitates the study of a complex construction through a single quantitative variable, it may present an issue for researchers who are looking to target a specific aspect of social experience, context or interaction. For example, in the Diveica et al. (2023) English socialness norms, some of the words with the highest ratings are *romance, socialism, mother, festival* and *funeral*, highlighting the breadth of representations that socialness as a variable can capture, whilst also revealing the acute differences that exist between the concepts in terms of the specific types of social information that is encoded—*festival* and *funeral* are both social events, but the semantics are normally quite contrastive in terms of the social information they represent. Thus, an alternative approach has been to investigate specific attributes that are socially relevant and can be theoretically defined, providing a more nuanced quantification of socially pertinent dimensions of representations.

One example of a socially relevant dimension being examined in this way is semantic gender, where participants are asked to rate the extent to which a word’s meaning is associated with feminine, masculine or neutral (i.e. neither feminine nor masculine) attributes. For example, in Vankrunkelsven et al. (2024), the word *vader* (father) is rated as masculine, the word *moeder* (mother) as feminine, and the word *knoop* (button) as neutral. Semantic gender was included as a scale in Osgood et al.’s (1957) original semantic differential work, which only focussed on a very small set of words (see Vankrunkelsven et al., 2024, for a more detailed overview of other studies that have normed semantic gender). However, with the renewed attention to collecting mega-study datasets, there are now conceptual/semantic gender ratings available for 5,500 (Scott et al., 2019) and 2,373 (Lewis et al., 2022) English words, as well as 24,000 Dutch words (Vankrunkelsven et al., 2024). Even though these datasets are still relatively new additions to the larger catalogue of norms available to researchers, they have provided a critical bridge between the social and cognitive sciences, facilitating novel investigations into multidisciplinary domains of research—for instance, by uncovering sources of gender biases present in children’s literature (Lewis et al., 2022) or language more generally (Lewis & Lupyan, 2020), in addition to testing effects of linguistic relativity, whereby grammatical gender may influence the way that semantic gender is represented (Brand et al., 2025; Vankrunkelsven et al., 2024).

Indeed, the field of sociolinguistics has long demonstrated the importance of socially meaningful variables that can be used to characterise groups of individuals, (e.g. gender, age, location, political alignment) to study language use, variation and change (see e.g. Labov, 2011; Trudgill, 1974). Whilst such variables are the focus for studies at the population level (i.e. which words or variants are produced by females/males or younger/older age groups), there has been relatively little work looking at how these factors operate at the semantic level (i.e. which words have meanings that are associated with femininity/masculinity or younger/older age groups). Interestingly, when studies have used stimuli that have been chosen because of their associations with socially relevant dimensions of meaning, the findings have helped to inform models of language and memory. For example, Walker and Hay (2011) demonstrated that lexical access to words that are socially skewed towards either younger or older individuals (e.g. *internet* and *libraries*) is faster when the word is spoken by an age-congruent voice, with these results extending to associations for gender and non-spoken stimuli, such as images (Hay et al., 2019).

Whilst gender has received some attention in the norming literature, there has also been interest in other socially relevant dimensions of meaning, but typically this has looked at the way individuals attribute those dimensions to specific social groups, to better understand social attitudes and stereotyping. For example, semantic differential scales have been used to investigate whether there are differences in the way older and younger age groups are perceived. Rosencrantz and McNevin (1969) devised the Aging Semantic Differential, where participants were tasked with selecting a point on bipolar scales that were anchored by a series of contrasting adjective pairs, e.g. optimistic/pessimistic. Participants were asked to use the scales based on their perceived associations towards younger, middle-aged and older groups of adults, with the design being adapted into different languages and administered to different demographic groups (see Ayalon et al., 2019, for review).

Yet, in the semantic norming literature, efforts have largely been concentrated on looking at age as a demographic variable that can explain variation in ratings, e.g. the extent to which some words are rated differently for emotional dimensions of meaning (Grandy et al., 2020) and not as a semantic variable that can be associated with concepts. To our knowledge, there have only been two studies that have more directly assessed the extent to which words are associated with age—one on German by Grühn and Smith (2008) and another on English by Lin et al. (2017). Both of these studies focussed on adjectives and assessing the extent to which participants associated the meaning of those adjectives with young or old age groups using a bipolar rating scale, with data from young and old participant groups. Although limited in the number and diversity of linguistic stimuli, with both studies reporting data on ~ 200 adjectives, they do offer novel insights into the way age is embedded in semantic meaning and how those associations can be quantified.

Ratings have also been used in research on political attitudes. Roberts and Utych (2019) examined how the relationship between gender and affective ratings is influenced by the gender and ideology of speakers. They found that words associated with masculinity correlate positively with dominance, but negatively with positive valence. Moreover, such words tend to appear more frequently in the language of Republican politicians than in that of Democratic politicians. This pattern aligns with the traits favoured by their respective voter bases (Clifford, 2020).

There is also ample research dedicated to studying the urban/rural divide, especially in terms of political attitudes. Several studies show that rural areas tend to be linked with more conservative attitudes and less trust in government, while urban areas tend to be more liberal and progressive (e.g. Kenny & Luca, 2021; Luca & Kenny, 2024; Luca et al., 2023). This division is present not only in the attitudes of people living in urban/rural areas, but also in how the urban/rural divide is represented in society. For example, Pospěch et al. (2024) analyse how urban and rural concepts are represented in Czech mass media, and show that they often appear in combination with the concepts of strength/weakness, laziness/diligence and being in/out of touch with reality. Using the word ratings to determine whether words strongly associated with urban/rural environments also tend to be strongly associated with liberal/conservative political attitudes or which concepts in general tend to be highly associated with urbanicity/rurality could further fuel this line of research.

Taken together, there is growing evidence that semantic variables that are linked to socially meaningful attributes—or socio-semantics—are important features of the human conceptual system. Although attention has been given to the dimension of semantic gender, this is only one possible source of socio-semantic information, and there are several other dimensions that are currently underexplored. By investigating multiple, distinct dimensions of social semantic information—e.g. not just looking at a broad definition of socialness, but instead inspecting different facets of that concept—we have the potential to uncover a more nuanced inspection of the contribution of these variables to theories of semantic representation. For example, the dimension of gender may encompass certain aspects of social roles, location associations may relate to social spaces, political alignment may underpin social ideologies, and age may be directly related to certain social characteristics.

Moreover, when there are datasets that quantify social dimensions, they tend to look exclusively at orthographically presented word forms, which substantially limits researchers who might want or need to design experiments that do not use linguistic stimuli. Therefore, if the study of socio-semantics is to advance, more coverage is needed in terms of the types of dimensions and the types of stimuli available as norms.

### The SocioLex-CZ norms

In this paper we introduce the SocioLex-CZ norms, the first large-scale dataset that quantifies socio-semantic representations across four distinct dimensions: gender, location,^1^ political alignment and age. Additionally, the dataset contains information on an affective dimension—valence—which was included because there are several existing studies with norms available, and it can act not only as a new dataset for Czech, but also as a control variable to facilitate our reliability analyses.

Across two experiments, we collected data for a diverse range of 2,999 words in Czech (Experiment 1), with an exploratory analysis of how the dimensions relate to each other, as well as how they compare to existing datasets in other languages. We also collected data for 1,000 images (Experiment 2), with another exploratory analysis of how the different stimulus types may modulate the ratings.

The primary motivation for developing the SocioLex-CZ dataset is to provide researchers with a novel resource that captures the semantic representations of stimuli along several theoretically meaningful social dimensions for both words and images. Moreover, this is the largest dataset to examine speakers of the Czech language, opening up new possibilities for researchers interested in studying a language with a rich morphology and a distinctive recent geopolitical history.

The following sections introduce the specific dimensions of interest and the methodology used for data collection and data pre-processing in the present study. We then describe and validate the summarised dataset of normative ratings and explore the relationships between the different dimensions and the different stimuli versions (words and colour/grayscale images). We also assess the extent to which these norms can predict lexical processing times. Lastly, we outline future directions and applications for the study of socio-semantics.

## Experiment 1: Word ratings

Following the procedure in previous studies (e.g. Vankrunkelsven et al., 2024), we collected ratings from a large participant sample for a wide range of Czech words, using five different socially relevant dimensions (gender, location, political, valence and age).

### Methods

#### Stimuli

The stimuli consisted of 2,999 written words (1,902 nouns, 766 adjectives and 331 verbs). The word list was constructed with the aim of including items that refer to a broad range of socially relevant and culturally salient concepts, across a range of different parts of speech. This was achieved by exploring word lists from published datasets. First, we took all 501 Czech word labels from the Multilingual Picture Database (Duñabeitia et al., 2022), which were the most common names for coloured drawings of everyday objects. Second, we took all 822 Czech role nouns used in Misersky et al. (2014), which focussed on gender stereotypicality. Third, we took Czech translations of the Leipzig–Jakarta list (Haspelmath & Tadmor, 2009), which contains 100 basic cultural concepts. Fourth, we took 400 translation equivalents of German adjectives from Grühn and Smith (2008), which focussed on a range of semantic rating scales, including age. Fifth, we took the 300 most frequent verbs from the Czech National Corpus SYN2020 (Jelínek et al., 2021), which consists of contemporary written Czech. Finally, we included the remaining 876 items during a brainstorming session between the researchers.

Since Czech is a grammatically gendered language, both masculine and feminine variants were included whenever possible. This concerned mostly role nouns (e.g. *pekař/pekařka*, which refer to male/female baker), nouns referring to animals (e.g. *medvěd/medvědice*, which refer to male/female bear) and adjectives (e.g. *dřevěný/dřevěná*, which are adjectives meaning *wooden* and are used respectively for grammatically masculine/feminine heads in a phrase). Nouns and adjectives were presented in nominative singular, whereas verbs were in their infinitive form.

The list of words was pseudo-randomly divided into 100-word subsets. Each subset contained approximately the same number of nouns, adjectives and verbs. We also controlled the distribution of grammatically gendered words by ensuring no subset contained both grammatically feminine/masculine variants of the same word, with the number of grammatically feminine/masculine adjectives and nouns being roughly comparable across lists. All lists were further complemented with four phonotactically plausible pseudowords (e.g. *tontota*), which served as control words for an attention check, as well as a calibrator word for each socio-semantic dimension, which was always the first word presented in the list. The decision to include only a single calibrator word for each dimension was to ensure that the experiment timing was kept reasonable, although we acknowledge that more calibrator words would have been optimal. The calibrator words were chosen on the basis of data from a pilot experiment, whereby participants reliably rated the words towards a specific direction on the scale. The calibrator words were gender – *náhrdelník* (necklace), location – *metro* (subway/underground train), political – *tradice* (tradition), valence – *šikanovat* (to bully), age – *důchod* (pension).

#### Participants

An initial sample of 1,475 participants completed the experiment, of which 1,275 participants were recruited from a university-wide student participant pool at Charles University in the Czech Republic, with all students receiving course credit for taking part. Ethical approval was given by Charles University. From this sample, we excluded 48 participants who reported that their native language was not Czech. An additional 200 participants were also recruited using Prolific (www.prolific.com), who were paid £5.00 for taking part and declared that they were currently a university student and had Czech as their first language (L1). We also decided to only retain participants who reported their age as between 18 and 30 years, which meant excluding 128 participants altogether. The motivation for restricting the age was to ensure our dataset would be comparable to other existing datasets with predominantly young adults (e.g. Vankrunkelsven et al., 2024). After a series of data quality checks (see ‘Data cleaning’ section below), we excluded a further 45 participants, resulting in a final sample of 1,254 participants (936 female, 312 male, 6 non-binary/self-report, median age = 21, *SD* = 2.22, range = 18–30).

**Fig. 1:**
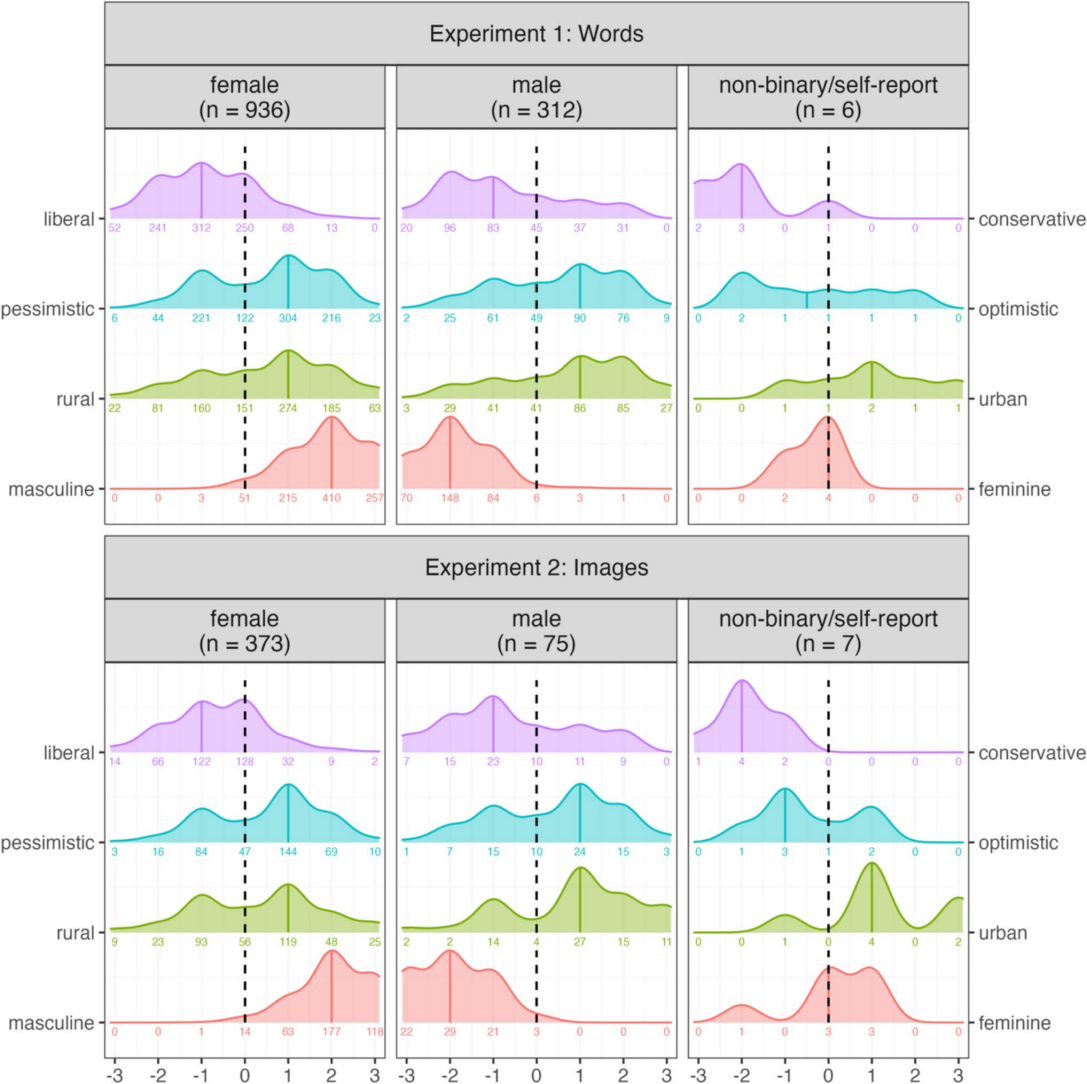
Demographic profile of participants from Experiments 1 and 2. Facets are used to separate the data based on categorical gender, with the number of participants given in brackets. The y-axis separates the different demographic questions. The values on the x-axis represent a numeric transformation of the seven-point Likert scale, i.e. − 3 represents very liberal/pessimistic/rural/masculine, 3 represents very conservative/optimistic/urban/feminine, and 0 represents the neutral midpoint. Counts of participants who selected each scale point are given as numbers under each density plot. For example, in Experiment 1, 52 out of the 936 females identified as very liberal

All participants completed a demographic questionnaire before starting the experiment. This was designed to collect a more detailed demographic profile of the participants and included standard questions, e.g. age, categorical gender (female/male/non-binary/self-report), highest educational qualification and native language, but also questions about their perceived socio-demographic profile. This included seven-point Likert scales where participants self-assessed their gender stereotypicality (typical male–typical female), character (very optimistic–very pessimistic), location affiliation (very urban–very rural) and political alignment (very liberal–very conservative). All scales were bipolar, with a neutral midpoint. These data are visualised in Fig. 1. We include these variables in the OSF repository, but note that the aims of this paper are not focussed on providing a detailed analysis of how demographic differences may influence ratings. We return to this in the general discussion and address this in ongoing work where sufficient depth and attention can be given.

#### Procedure

The experiment was designed and distributed online using Qualtrics (https://www.qualtrics.com). Participants first read a detailed instruction page, provided informed consent and completed the demographic questionnaire described above. The original Czech and translated versions are available in the OSF repository. Participants were then asked to rate each of the words from a randomly selected list (each containing 100 words, one calibrator word and four pseudowords). Specifically, participants were asked to rate the words based on how they associated the meaning of the word according with each of the following dimensions:

- gender (scale anchors: very masculine–very feminine). Czech version: gender (*silně mužské–silně ženské*).
- location (scale anchors: very rural–very urban). Czech version: prostředí (*velmi venkovské–velmi městské*).
- political (scale anchors: very liberal–very conservative). Czech version: politické přesvědčení (*velmi liberální–velmi konzervativní).*
- valence (scale anchors: very positive–very negative). Czech version: emoce (*velmi pozitivní–velmi negativní*).
- age (scale divided into categories of 0–6, 7–17, 18–30, 31–50, 51–65, 66–80 and 81 + years). Czech version: věk.

**Fig. 2:**
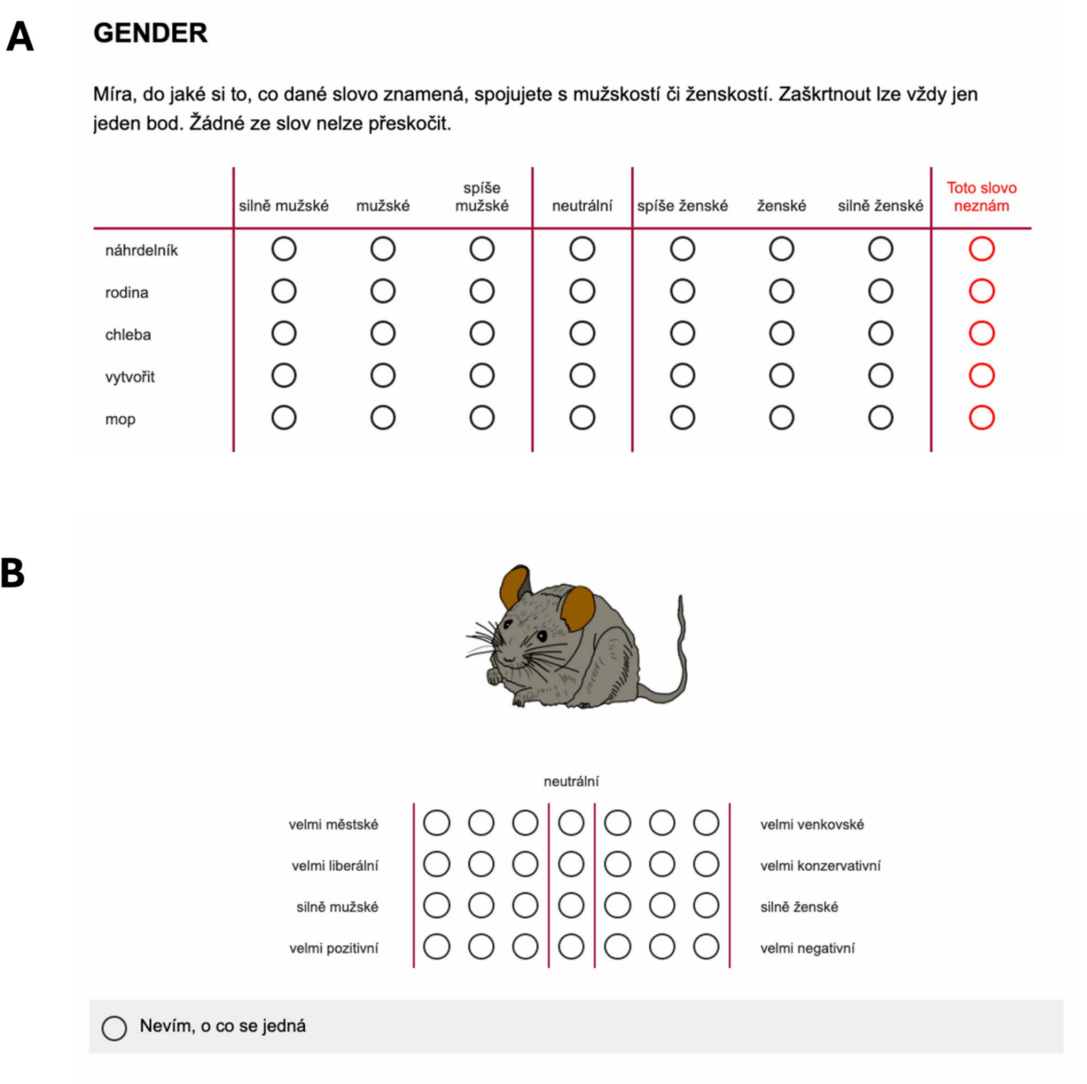
Examples of the rating procedure used in Experiment 1 (***A***) and Experiment 2 (***B***). The translation of the instructions in ***A*** is ‘The degree to which you associate the word meaning with masculinity or femininity. Only one point can be ticked at a time. None of the words can be skipped.’ The anchors of the scale (from left to right) translate to ‘very masculine/masculine/slightly masculine/neutral/slightly feminine/feminine/very feminine’

The instructions given to participants for the rating task, as well as Qualtrics files and pdf versions of the full experiment in Czech (and their English translation), can be found in the OSF repository. Participants were presented with one dimension at a time, which contained all the words from one of the subsets. The order of presentation for dimensions and words was randomised for each participant (apart from the calibrator word, which was always presented first, and the age dimension, which was always presented as the last dimension). All dimensions (apart from age) were rated using seven-point Likert scales, each with a neutral midpoint; see Fig. 2A. For the age dimension, participants were instructed to select as many age categories as they felt were applicable using checkboxes, meaning multiple categories or no categories could be chosen. This was done so that participants could provide a more detailed age selection that was not restricted to just one forced choice; e.g., the word *pensioner* would likely activate both 66–80 and 81 + categories, whereas *paper* would activate no categories. All dimensions had the option to skip a word if the meaning was not known (*toto slovo neznám*).

### Data cleaning

All cleaning, processing and analyses reported below were conducted using R version 4.3.1 (R Core Team, 2023). All the code and data (raw and processed) can be found in the OSF repository.

**Fig. 3:**
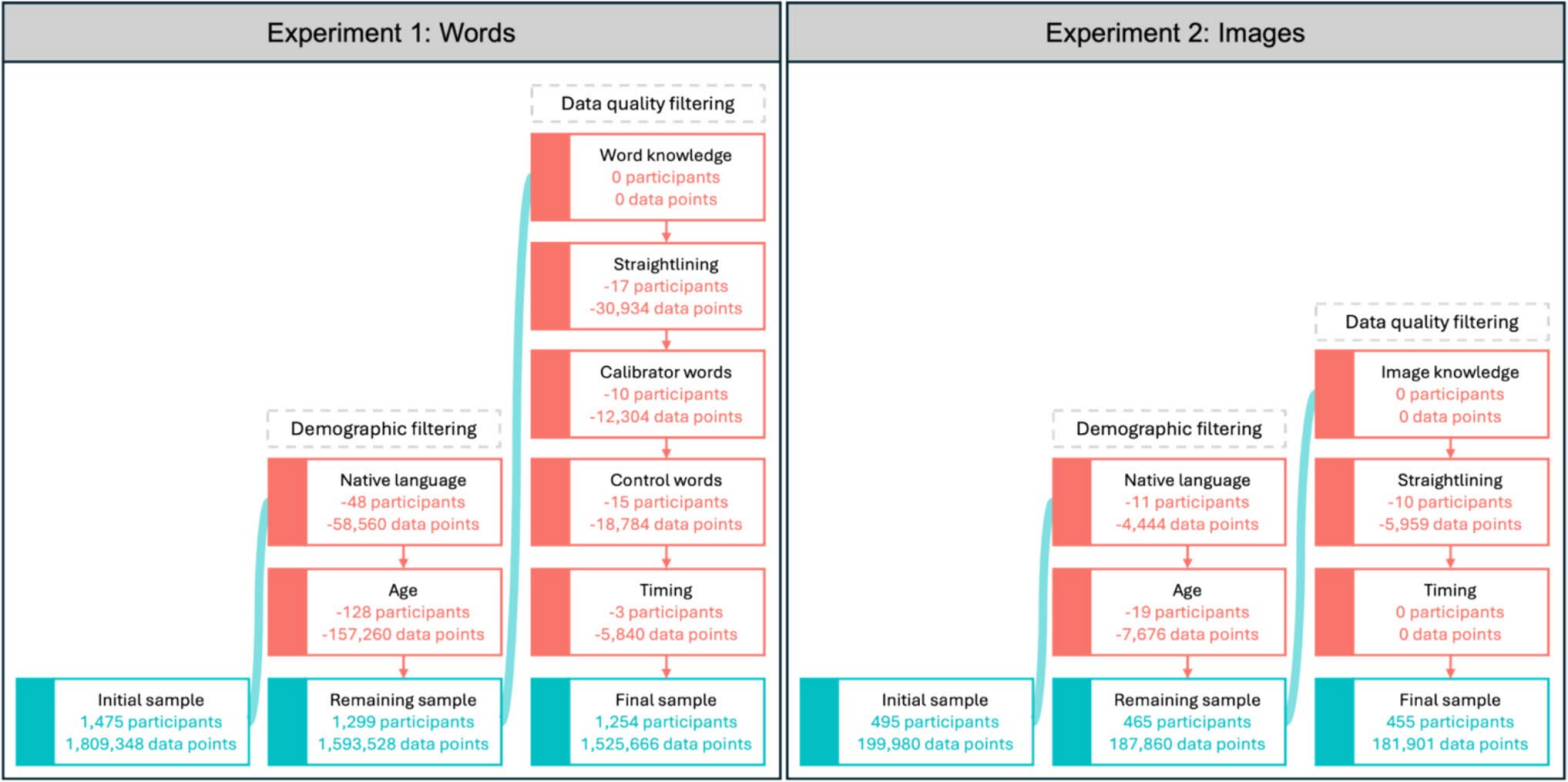
Filtering procedure applied to the data from Experiments 1 and 2. Turquoise boxes contain summaries of the data that remain after each filtering block; red boxes contain summaries of the data removed during each filtering step

We carried out a number of data quality checks to ensure that we detected any low-quality responses/participants; this process is visualised in Fig. 3. The code used to run these checks can be found in the OSF repository.

1. *Word knowledge:* We checked for any participants who reported not knowing over 20% of the words they were presented with; however, there were no such participants.
2. *Straightlining:* We inspected the variance in the participants’ responses to identify straightliners, i.e. participants who selected the same response for more than 80% of the items (Kim et al., 2019; Winter et al., 2024). If a participant straightlined on more than three out of the five dimensions, we decided not to include them in the final dataset, resulting in the exclusion of 17 participants. Additionally, we removed a participant’s response to words on a specific dimension if they straightlined for over 95% of the words in this dimension, resulting in the exclusion of 31 responses.
3. *Calibrator words:* We analysed the responses to the calibrator words for each of the five dimensions to ensure that participants could demonstrate that they were using the rating scales as expected. For each calibrator word, we identified participants who responded to the word in the opposite side as expected. For example, the word *metro* for the location dimension was expected to have a response of urban, so if a participant responded to this word as rural in any way (i.e. slightly rural, rural or very rural), this would be flagged. Any participant who responded to two or more calibrator words in the opposite direction to our original expectations would be excluded, resulting in the exclusion of 10 participants.
4. *Control words:* We also analysed the responses to the control words (pseudowords that were phonotactically legal in Czech), excluding any participant who consistently rated all four of these words throughout the experiment instead of choosing the ‘*I don’t know this word*’ option. This resulted in the exclusion of 15 participants.
5. *Timing:* We inspected the time it took for each participant to complete the ratings along each of the dimensions. We first transformed the timings to natural log values, then calculated the mean and standard deviations for each of the dimensions. A participant was then excluded completely if they were quicker than the mean by 2.5 times the standard deviation for two or more dimensions (this was always less than 116 s, with the mean values ranging between 349 and 451 s). This resulted in the exclusion of three participants. Additionally, we removed a participant’s response to words on a specific dimension if their timing was quicker than the mean by 2.5 standard deviations, but retained all their other responses, resulting in the removal of data for 13 responses to individual dimensions. There was no upper cut-off point, since no time constraints were mentioned in the instructions and participants could take however long they needed to finish the experiment. Typically, participants took around 6 min to complete their ratings on each dimension, with the maximum time spent being ~ 17 min.

### Data summaries

We processed the raw data in a number of different ways to generate the final summary data for the SocioLex-CZ norms. We processed the dimensions of gender, location, political and valence differently from the age dimension, as the scales differed.

#### Gender, location, political and valence

##### Descriptive statistics

**Fig. 4:**
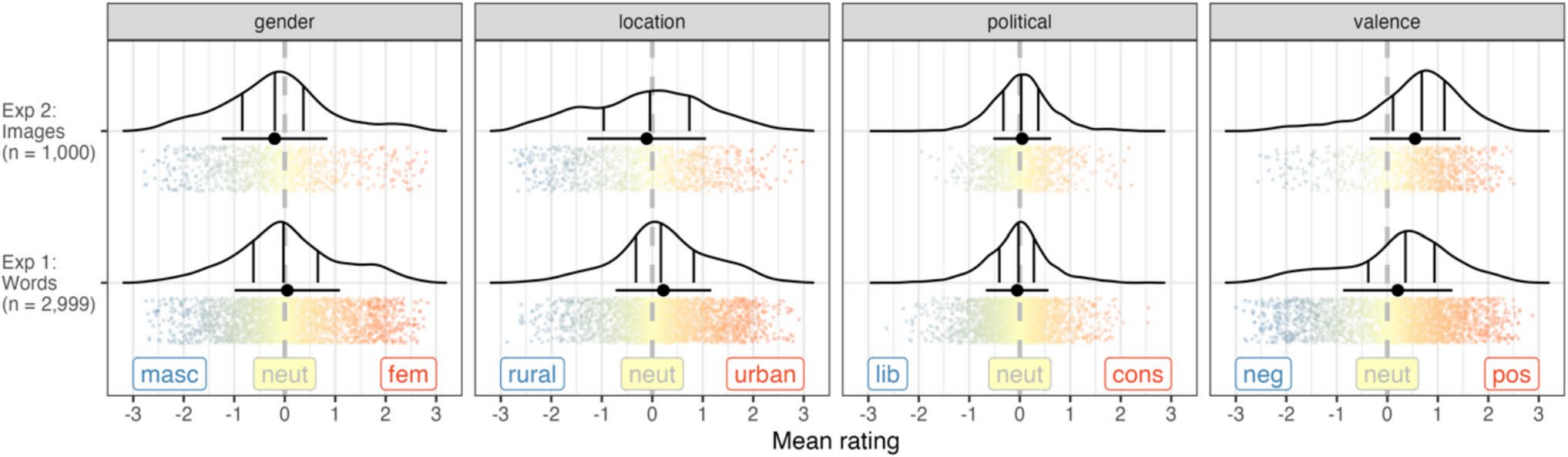
Distribution of mean ratings for the gender, location, political and valence dimensions for Experiments 1 and 2. Kernel density estimates are shown with 25%, 50% and 75% quantiles marked by solid vertical lines, with the dashed line representing a value of 0 (neutral). The black point with a horizontal line represents the mean and standard deviation of the ratings. Each word is represented by a point, with more red/blue colours indicating a stronger association towards a specific side of the scale, and yellow points associating more with neutral ratings

For the dimensions of gender, location, political and valence, the values were first transformed to numeric scales, ranging from − 3 (very masculine/rural/liberal/negative) to 3 (very feminine/urban/conservative/positive), with 0 being the neutral midpoint. Responses where the participant indicated that they did not know the word were coded as NA values. From these data we calculated the mean, *SD*, number of responses overall, number of responses where the word was known, and the proportion of known responses for each of the words across the dimensions; see Fig. 4 for a visualisation of the data and Table 1 for sample size summaries. We can see that the majority of ratings are around the neutral midpoint, with a skew towards more positive ratings for the valence dimension, which has also been reported in other studies (e.g. Warriner et al., 2013). Table 2 gives examples of the items with the most extreme values for each dimension.

**Table 1:** Summary of the number of responses to items across the two experiments

Experiment	Median *N*	Range *N*	Median *N* known	Range *N* known	Mean prop known	Range prop known
Exp 1: Words	37	[28, 96]	37	[11, 96]	.997	[.35, 1]
Exp 2: Images	45	[35, 52]	45	[10, 52]	.978	[.26, 1]

**Table 2:** Items with the most extreme values for each of the dimensions available in the dataset. Items are given in Czech with their associated values (means or PC scores) in brackets. See the OSF repository for English translation equivalents

Dimension	Items	
gender	Masculine	*otec (− 2.74), varlata (− 2.71), táta (− 2.69), muž (− 2.67), Vladimír (− 2.67), penis (− 2.62), mužský (− 2.61), strýc (− 2.59), Rudolf (− 2.55), Adam (− 2.53)*
	Feminine	*Božena (2.83), princezna (2.75), vagina (2.75), Sofie (2.7), Adéla (2.68), podprsenka (2.68), matka (2.65), Karolína (2.64), Viktorie (2.63), žena (2.62)*
location	Rural	*venkov (− 2.88), orat (− 2.62), venkovská (− 2.59), vesnice (− 2.59), farmářka (− 2.59), traktor (− 2.58), farma (− 2.55), chalupa (− 2.4), stodola (− 2.39), venkovský (− 2.38)*
	Urban	*Praha (2.91), velkoměsto (2.78), metropole (2.78), mrakodrap (2.77), město (2.74), Berlín (2.74), New York (2.72), fastfood (2.71), Vídeň (2.68), Londýn (2.68)*
political	Liberal	*liberalismus (− 2.64), liberální (− 2.3), transgender (− 2.24), bisexuálka (− 2.18), multikulturalismus (− 2.15), bisexuál (− 2.14), lesba (− 2.13), transsexuál (− 2.12), veganka (− 2.12), transsexuálka (− 2.07)*
	Conservative	*konzervativní (2.55), konzervatismus (2.54), monarchie (2.04), homofobie (1.97), církev (1.92), křeštanství (1.89), bible (1.88), království (1.85), šlechta (1.84), staromódní (1.83)*
valence	Negative	*znásilnění (− 3), znásilnit (− 2.84), rakovina (− 2.82), terorismus (− 2.82), násilník (− 2.78), terorista (− 2.73), pedofil (− 2.68), vrah (− 2.67), zmrd (− 2.67), nacismus (− 2.65)*
	Positive	*šťastný (2.88), zdravá (2.63), šťastná (2.62), láska (2.59), smát se (2.59), spokojenost (2.59), milovat (2.54), radost (2.53), svoboda (2.53), štěstí (2.51)*
age pc_1_	Old	*penze (− 5.68), stáří (− 5.66), seniorka (− 5.49), děda (− 5.4), babička (− 5.4), senior (− 5.31), stará (− 5.3), starý (− 5.23), alzheimer (− 5.19), stárnutí (− 5.17)*
	Young	*beďar (3.79), gymnázium (3.73), panictví (3.69), dospívání (3.6), selfie (3.52), opisovat (3.51), akné (3.48), škola (3.47), výtvarka (3.46), drzý (3.42)*
age pc_2_	Middle-aged	*statistik (− 3.72), rozvod (− 3.58), účetní (− 3.53), podnikání (− 3.47), dozorce (− 3.37), architektka (− 3.37), popelářka (− 3.35), byznys (− 3.34), exekutorka (− 3.33), manažer (− 3.3)*
	Young/old	*hřbitov (4.86), rakev (4.8), kremace (4.8), starý (4.51), zemřít (4.47), stará (4.4), pohřeb (4.4), smrt (4.34), senior (4.21), demence (4.12)*
age pc_3_	No age	*přehrada (− 3.31), Litva (− 2.89), tsunami (− 2.78), špičatý (− 2.74), Lotyšsko (− 2.61), Irsko (− 2.61), široká (− 2.57), voda (− 2.52), hmyz (− 2.51), hovězí (− 2.5)*
	Old	*senior (4.34), rakev (4.26), stáří (4.16), seniorka (4.14), starý (4.13), stará (4.11), hřbitov (4.08), penze (3.94), děda (3.83), kremace (3.72)*

##### Demographic-based variation

**Fig. 5:**
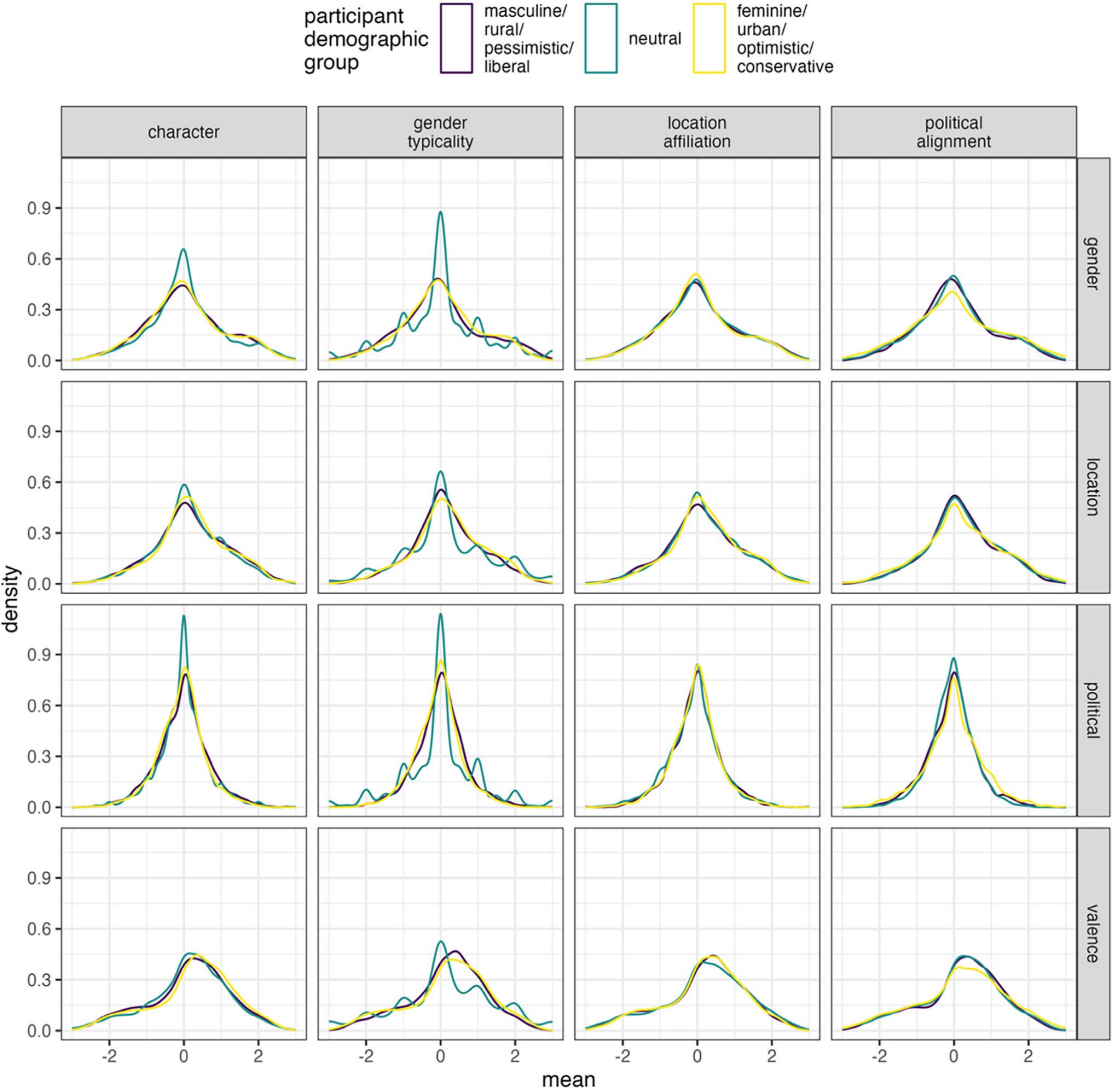
Distribution of mean ratings for the gender, location, political and valence dimensions for Experiment 1 based on participant socio-demographic categories. The x-axis shows the mean rating for the words, with the y-axis representing the kernel density estimates. The top panel of facets distinguishes the socio-demographic variables, whereas the side panel facets indicate the dimensions. The colours indicate the socio-demographic categories of participants

As we collected a number of different demographic variables that were self-reported by participants, we inspected whether there existed any underlying variation in the ratings for the stimuli that could potentially be explained by these demographic variables. However, as our participant sample was skewed towards certain demographic characteristics (see Fig. 1), we were limited in terms of looking at the variables in a detailed manner—i.e., it would be difficult to explore whether participants who identified as very conservative differed from those who identified as slightly conservative, as there were only a very limited number of participants in those categories. Therefore, we used the data from the self-assessed socio-demographic questions to categorise participants into three distinct groups for each question based on the side of the Likert scale they used, namely participant gender typicality (typically masculine or feminine), character (pessimistic or optimistic), location affiliation (rural or urban) and political alignment (liberal or conservative), with any neutral responses treated as an independent category. We then calculated the mean ratings for each of the participant groups for each of the stimuli, thus obtaining word profiles across the socio-demographic categories of participants. The results revealed very little difference across the demographic groups; see Fig. 5. This pattern is similar to the findings reported in Vankrunkelsven et al. (2024) and Preininger (2025), who also found that female and male participants did not show any major differences in the way they rated words for the gender dimension.

##### Proportions and entropy

We also provide additional descriptive statistics for each of the items that may be of interest to researchers. Specifically, raw counts are available for each item for each of the dimension’s scale points, allowing for a more nuanced description of how often each response was selected; these have also been transformed into proportional values. Additionally, we used the count data to calculate the Shannon entropy (Shannon, 1948) for each item, along each dimension, providing an alternative measurement to *SD*. Entropy was included because it captures uncertainty in the ordinal Likert scale, whereas *SD* is better suited to measuring variance in numeric scales.

##### Latent means

For aggregating rating data from Likert scales, many datasets have been published with the means for each item, where the ratings are treated as integer values, e.g. very negative on our valence scale as − 3. However, this approach may not be the most suitable, as means do not preserve the ordinal nature of the Likert scales being used (see Liddell & Kruschke, 2018; Veríssimo, 2021). Therefore, we followed the guidance of Taylor et al. (2023) and modelled the participant responses using cumulative link mixed-effects models with the ordinal package (Christensen, 2023) in R. These models account for variation introduced from the participant response biases, and thus provide a more accurate estimation of a normative estimate for each item. We modelled the ratings for each of the dimensions with a separate model, predicting the participant responses (coded as an ordinal factor, i.e. − 3 < − 2 < − 1 < 0 < 1 < 2 < 3) with random intercepts for item and participant.^2^ From this we were able to extract the random intercepts for each of the items, providing us with a numeric estimate of the latent mean. We inspected the correlation between the latent means and the standard means, with the two variables correlating almost perfectly (all *r*s >.99).

#### Age

To obtain a normative estimate from the age ratings (which were not collected using Likert scales, but instead using categorical checkboxes where a single option, multiple options or no option could be selected), we needed to apply a number of processing steps to generate the summary values.

##### Weighted proportions

We transformed the raw categorical responses to calculate a weighted proportional value for each of the age categories. This was based on the number of age categories selected by a participant for each item (1/*n*_selected_categories): if a participant selected two age categories for an item, each selected category would have a weighted rating of 0.5 and all others would have 0; if one category was selected, then the value would be 1; if all seven categories were selected, the value would be 1/7; if no categories were selected, then an additional ‘no age’ category would have 1. From the weighted values we could then calculate a weighted proportion for each of the resulting eight age categories within each item, by summing the weighted proportions and dividing that value by the number of participants who rated the item (excluding responses where the participant did not know the word). This gave us a value between 0 (no responses for the category) and 1 (all participants selected only the one category), which could be used as an estimate of the associative strength for an item in each of the age categories.

##### Mode category

On the basis of the proportion values, we were also able to calculate the mode age category for each item, i.e. the category that had the largest proportional value, which could be used as a categorical summary variable that captured the most likely age association, e.g. *školka* [kindergarten], which has an age mode of 0–6. If there were multiple mode categories for an item, the category was randomly selected from the possible categories, e.g. *mechanička* [female mechanic], which had mode values of 18–30 and 31–50, but was assigned 18–30. Since we also took into account instances where no categories were selected, the mode could take the form of ‘no age’.

##### Principal component analysis (PCA)

**Fig. 6:**
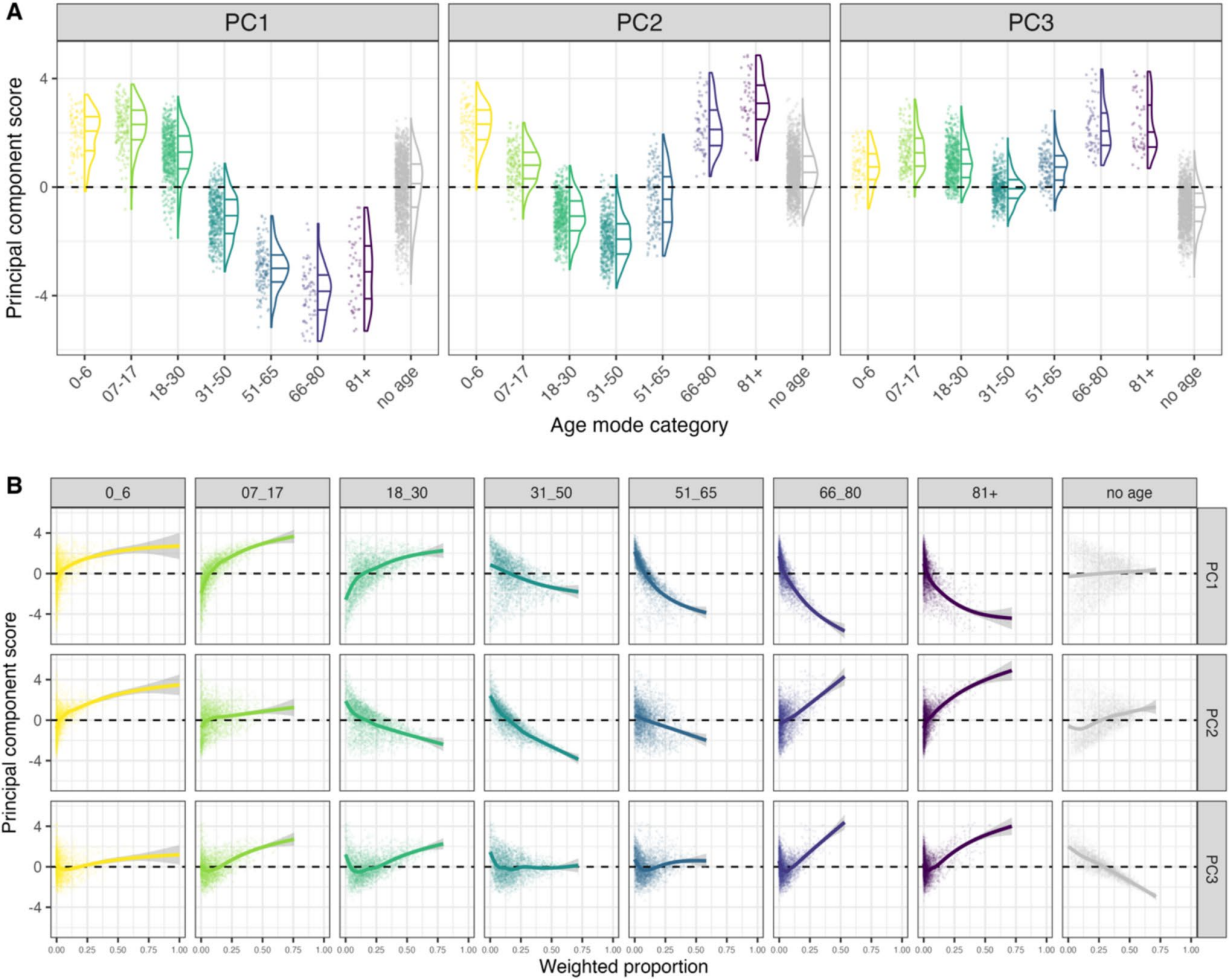
Visualisation of the results from the PCA for the age dimensions. **A** Distribution of PC scores (y-axis) based on the mode age of each word (x-axis) for each of the three PCs (facets). **B** The weighted proportions for each word (x-axis) based on the age categories (top facets) and their relationship to PC scores (y-axis) with a GAM smooth by PC (side facets)

To create a summary value for the age dimension, we used the weighted proportion values (where each row is an item and each column is an age category) as a 2,999 × 8 multidimensional space. This acted as the input data for a PCA, which allowed us to reduce the number of dimensions in the space by identifying the underlying structure in the data. The result of the PCA is a set of principal components (PCs) that can be interpreted based on how the original variables (age categories) are loaded, i.e. which variables co-vary together within each PC. Additionally, each item will have a principal component score that reflects the strength of association with each of the components, which will act as the individual item norm for each of the PCs (see Venables & Ripley, 2013; Brand et al., 2021; Wilson Black et al., 2023). The results of the PCA provided us with three main principal components (PC), each of which explained over 15% of the variance in the data and 74.19% of the overall variance. We will interpret the PCs individually as PC1, PC2 and PC3. The visualisations that can be used to guide the interpretation of the PCA results are presented in Fig. 6.

*PC1: Younger/older*. This PC accounted for 33.82% of the total variance and is interpreted as capturing the variation between age categories that are under the age of 30 (positive PC1 scores) or over the age of 30 (negative PC1 scores). We highlight that this is not a linear relationship, and the most extreme PC1 scores are not directly related to the youngest or oldest age categories, but instead represent a more general distinction between young and old associations; for example, *beďar* [pimple] has the highest PC1 score of 3.79, whereas *penze* [pension] has the lowest score of − 5.68.

*PC2: Middle-aged*. This PC accounted for 24.82% of the total variance and is interpreted as capturing the variation between age categories that are related to middle age and those which are not. This appears to be a U-shaped relationship, where negative values are most strongly associated with the 31–50 age category and more positive values with the youngest (0–6) and oldest age categories (66–80 and 81 +); for example, *statistik* [male statistician] has a PC2 score of − 3.72, whereas *hřbitov* [graveyard] has a score of 4.86.

*PC3: No age*. This PC accounted for 15.54% of the total variance and is interpreted as distinguishing between items that have no age associations and those that do. The negative PC3 scores represent stronger associations with no age, whereas positive scores appear to represent stronger associations with any age category, but specifically the oldest age categories; for example, *přehrada* [dam] has a PC3 score of − 3.31, whereas *senior* [male senior citizen] has 4.34. We note that the individual weighted proportion scores for the no age category would likely serve as a more accurate representation of this PC, as it can be interpreted more straightforwardly, whereas the PCA introduces more complexity when interpreting the positive values; see Fig. 6B.

### Analysis and results

#### Reliability analysis

The internal consistency of the individual rating scales was assessed using Cronbach's alpha (Cronbach, 1951), allowing us to measure the reliability of our item ratings—in this case, each of the socio-semantic dimensions. We calculated alpha values for each list of words^3^ individually for the gender, location, political and valence dimensions, where only one option per word could be selected. In the case of the age dimension, participants could choose as many options as they liked, which yielded a large variance in individual ratings, rendering the calculation of Cronbach’s alpha unsuitable. The range of alpha values across the lists were as follows: gender = [.92,.95], location = [.89,.95], political = [.88,.95], valence = [.90,.95]. This indicates that the rating scales can be considered to have high reliability.

#### Correlations between variables

**Fig. 7:**
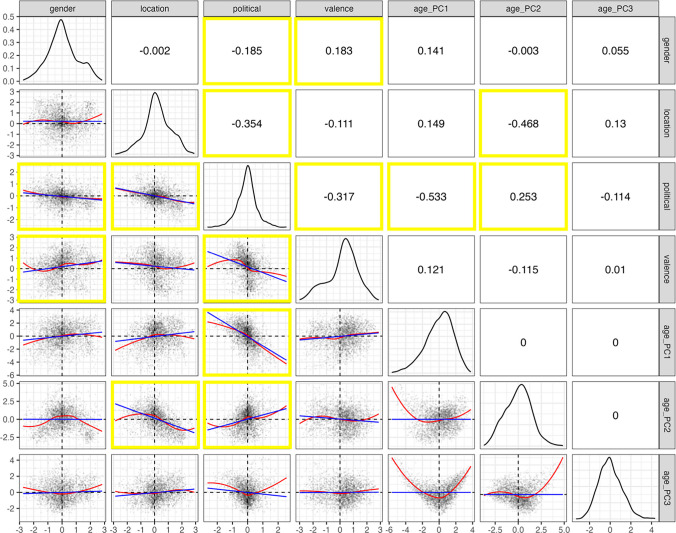
Correlation matrix of all the dimensions in the word ratings dataset. The lower left plots are scatter plots of the data fitted with a linear (blue) and loess (red) fit, with the data from the dimension in the top facets on the x-axis and the data from the right facet on the y-axis. The diagonal plots show kernel density distributions of the variables. The upper right plots give the Pearson correlation coefficients between two dimensions. Notable correlations (r >|.18|) are highlighted by yellow outlines

We ran an exploratory correlational analysis to inspect whether the mean values for each item were correlated across the different socio-semantic dimensions. As the means across all the dimensions had a reasonably normal distribution, we conducted Pearson’s correlations between each of the dimensions; see Fig. 7 for the visualisation of the results. Note that as this was an exploratory analysis and we were not directly testing any research hypotheses, we purposefully do not report *p* values from the tests and will instead simply describe patterns in the data where the correlations were strongest, i.e. *r* >|.18|.

The strongest correlation was between political and age pc_1_ (*r* = −.533), indicating that liberal items were more likely to be associated with youth, and more conservative items with older age. political was also related to location (*r* = −.354: more liberal items are associated with urban environments, conservative with rural), valence (*r* = −.317: more liberal items are associated with positive valence, conservative with negative), age pc_2_ (*r* =.253: more liberal items are associated with middle age) and gender (*r* = −.185: more liberal items are associated with femininity, conservative with masculinity). location was also correlated with age pc_2_ (*r* = −.468), indicating that more urban items were associated with middle age. gender and valence were also correlated (*r* =.183), with more feminine items associated with positive ratings, whereas masculinity was associated with negative ratings, which was also reported by Scott et al. (2019) for English data (the authors collected both gender and valence ratings) and by Vankrunkelsven et al. (2024) and Moors et al. (2013) for Dutch data (based on their gender ratings and Dutch valence ratings from Moors et al., 2013).

We would like to highlight that not all of these relationships were linear; indeed, in Fig. 7 we fitted a loess smooth to the data to demonstrate this more nuanced interpretation of how the dimensions may relate to one another, especially at the more extreme ends of the distributions. However, a more detailed examination of these relationships is outside the scope of the current paper.

#### Correlations with other languages

As an additional level of analyses, we were interested in looking at how our data correlated to other existing datasets with the same underlying dimensions, testing the hypothesis that our data would correlate with other datasets in different languages. As this is the first large-scale norming dataset of variables for Czech words, we unfortunately could only assess whether our dataset correlated to existing datasets in other languages, instead of other datasets in Czech. We included the valence dimension in our norms, as the valence norms for English words by Warriner et al. (2013) have been used extensively by researchers since they were published, and offered a good baseline for comparison. Additionally, Scott et al.’s (2019) dataset includes both valence and gender norms for English words, which provided us with an additional resource that was published more recently.

Subsequently, we coded all 2,999 Czech words for translation equivalents in English (which were translated and checked by two native Czech speakers, both highly proficient in English), providing us with a basis to compare across the different datasets. We then filtered the data so that we had the original Czech mean ratings for valence and the mean ratings of the translation equivalents in English. Using this dataset, we ran Pearson’s correlations to inspect the relationship between the Czech and the English ratings. From Warriner et al.’s (2013) valence ratings, we had 1,546 comparable items, which demonstrated a very strong correlation, *r*(1544) =.873, *p* <.001. We took both the valence and gender ratings from Scott et al. (2019) with 1,166 comparable items and again found a very strong correlation for valence, *r*(1,164) =.916, *p* <.001, and a strong correlation for gender, *r*(1,164) = −.658, *p* <.001.^4^ These results demonstrate that even though there may be cross-linguistic differences in the word forms used across different languages, there is strong support for the hypothesis that, across the dimensions of valence and gender, these differences do not result in large differences in the associations perceived by participants, although we do note that a more diverse cross-linguistic comparison could lead to nuanced differences.

#### Predicting processing times in a lexical decision task

In order to provide an additional measure of validity, we also analysed the extent to which the socio-semantic variables were predictive of measures of lexical processing. To do this, we used data from a large-scale lexical decision task by Brand et al. (in prep), which has data for ~ 10,000 Czech words, and the participants responding to each word matched the demographic profile of participants in the present study (the median number of participants responding to each item was 66), in addition to normative ratings for concreteness and age of acquisition (AoA) using methodological designs that aligned with Brysbaert et al. (2014) and Kuperman et al. (2012), respectively (again, the participant sample matched the demographics in the present study, with a median of 29 participants ratings each item). We also extracted word frequencies derived from Czech subtitles using data from Van Paridon and Thompson (2021), and calculated the orthographic word length for each of the unigram words in our dataset. The frequency data and reaction times for accurate responses in the lexical decision data were log-transformed, and any items in our word list without a frequency estimate or response time were removed (*n* = 190). The final dataset comprised 2,809 items.

We then conducted a hierarchical regression analysis on this dataset, predicting the log reaction times of items. We decided not to conduct an analysis on accuracy data from the lexical decision task, as the mean proportion of accurate responses on the items was 98.37%. The first step was to include linguistic and semantic variables that had previously been reported to be reliable predictors of lexical processing—frequency, length, AoA, concreteness and valence (see e.g. Diveica et al., 2023). The second step included the socio-semantic predictors of gender, location, political, age pc_1_ and age pc_2_. As we did not have any theoretical reason to expect the variables of gender, location and political to have a directional effect—i.e. we did not expect to find a linear relation wherein words that were more feminine were processed faster than words that were more masculine—we transformed the ratings from these variable to represent *ladenness*—the absolute rating value where ratings at the extreme ends of the scale were taken as larger values and ratings around the neutral midpoint were taken as smaller values. Thus, items that were higher in ladenness would be expected to encode stronger socio-semantic representation, and therefore be more likely to predict processing times, in line with theoretical accounts that predict a role of social information in processing (Barsalou, 2020; Borghi et al., 2019). We had no specific predictions about the age dimensions, as these variables do not necessarily represent ladenness given that the dimensions capture a continuity between either young and old (age pc_1_) or young/old and middle-aged (age pc_2_).

**Fig. 8:**
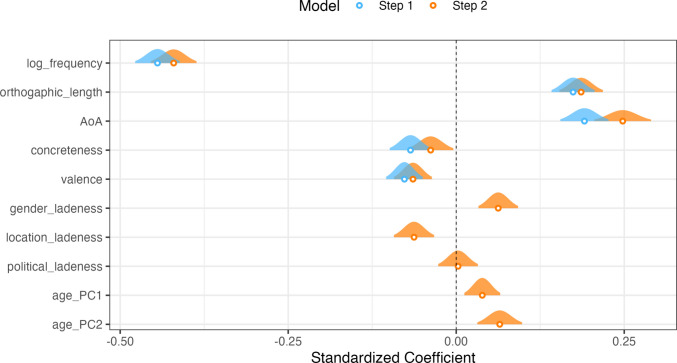
Regression coefficients visualised based on the step of the hierarchical regression modelling, where log reaction times from a lexical decision task are predicted by lexical and psycholinguistic predictors in Step 1 (blue) and with socio-semantic predictors added in Step 2 (orange). The distributions show 95% confidence intervals; standardised coefficients are on the x-axis, and the predictor variable names are shown on the y-axis

The results of the analysis are visualised in Fig. 8 and also summarised in Table 3. In Step 1, all of the predictor variables demonstrated a significant effect, predicting lexical times in a way that high-frequency, shorter-length, earlier-acquired, concrete and positive items were processed faster than items that were less frequent, longer, later acquired, abstract and negative. In Step 2, a significant improvement was obtained in model fit, *F*(5, 2798) = 11.393, *p* <.001, ∆*R*^2^ =.01. Although the increase in variance explained is relatively small (Step 1 *R*^2^ =.495; Step 2 *R*^2^ =.505), it is in line with other studies where additional semantic predictors were included (e.g. Diveica et al., 2023; Lynott et al., 2020). Tests for collinearity revealed no issues, with all variance inflation factors < 2.63.

**Table 3:** Summary of the hierarchical regression results from Experiment 1

Predictor	β	SE	*t*	*p*	*R*^*2*^	∆*R*^2^
**Step 1**					0.495	
Intercept	6.776	0.024	280.52	<.001		
Log frequency	− 0.083	0.003	− 26.31	<.001		
Orthographic length	0.014	0.001	10.63	<.001		
AoA	0.012	0.001	10.41	<.001		
Concreteness	− 0.012	0.003	− 4.30	<.001		
Valence	− 0.012	0.002	− 5.55	<.001		
**Step 2**					0.505	0.010
Intercept	6.711	0.026	253.85	<.001		
Log frequency	− 0.078	0.003	− 24.26	<.001		
Orthographic length	0.014	0.001	11.23	<.001		
AoA	0.015	0.001	11.50	<.001		
Concreteness	− 0.007	0.003	− 2.21	0.027		
Valence	− 0.010	0.002	− 4.48	<.001		
Gender ladenness	0.017	0.004	4.16	<.001		
Location ladenness	− 0.018	0.004	− 4.11	<.001		
Political ladenness	0.001	0.001	0.19	0.854		
Age PC_1_	0.004	0.001	2.85	0.004		
Age PC_2_	0.008	0.002	3.81	<.001		

We observed an effect for location ladenness, where items with a higher rating for either urban or rural association were processed faster than those that were neutral. This would suggest that these items may be processed faster as a result of the information they encode relating to social spaces. However, we observed a surprising effect for gender ladenness, whereby items that had stronger associations with femininity or masculinity were processed more slowly than items having more neutral associations. Future work could further investigate the role of frequency in predicting such patterns; for example, *menstruace* [menstruation] is less frequent but heavily gender-laden and was processed more slowly than a neutral but more frequent item, e.g. *ananas* [pineapple]. An alternative route would be to explore quadratic effects, whereby items with more extreme gender ladenness are processed faster than those with moderate gender ladenness, which may have been obscured with a linear regression analysis. We did not observe an effect for political ladenness, which may again be explained by a frequency effect for items that are related to politics e.g. *multikulturalismus* [multiculturalism]. For the age dimension, we observed significant effects for both variables, with words relating to older items being processed faster than those for younger items for age pc_1_, whereas for age pc_2_ we observed that items more strongly associated with middle age were processed faster than items associated with young/old age categories.

## Experiment 2: Image ratings

Typically, semantic norms are only published for items that are represented orthographically, i.e. as words. However, we also wanted to provide socio-semantic norms for image stimuli, to allow researchers to utilise the resource in a wider range of experimental settings, e.g. tasks that might want to avoid text-based stimuli. Thus, we also provide an additional dataset of socio-semantic norms relating directly to image stimuli in colour and grayscale.

### Methods

#### Stimuli

Our stimuli consisted of 1,000 images taken from the Multilingual Picture Database (Duñabeitia et al., 2022). This database contains drawings for commonly encountered nouns from a range of semantic categories (e.g. role nouns, animals, tools, clothing), with naming data from a number of different languages, including Czech. The Multilingual Picture Database contains 500 unique images, but we decided to include both colour and grayscale versions of each image in our stimuli (providing us with 1,000 images in total). This decision was motivated primarily by the need for diverse stimuli sets that could be used in experimental designs that may require exclusively colour/grayscale stimuli.

#### Participants

An initial sample of 495 participants completed the experiment. All participants were recruited from a university-wide student participant pool at Charles University in the Czech Republic, with all students receiving course credit for taking part. From this sample, we excluded 11 participants who reported that their native language was not Czech. We again decided to retain only participants who reported their age to be between 18 and 30 years, which meant excluding 19 participants. After a series of data quality checks (see ‘Data cleaning’ section below), we excluded a further 10 participants, resulting in a final sample of 455 participants (373 female, 75 male, 7 non-binary/self-report, median age = 21, *SD* = 1.95, range = 19–29). All participants completed the same demographic questionnaire as described in Experiment 1, with the data visualised in Fig. 1.

#### Procedure

The procedure roughly matched the procedure for Experiment 1, but with a small number of changes. Participants were presented with one image at a time and were asked to rate the image along the dimensions of gender, location, political and valence using the same seven-point Likert scales; see Fig. 2B. The age dimension was not included for this experiment as it was not possible to incorporate the checkbox input required for this variable in the design using Qualtrics. Participants could also select an option (*nevím, o co se jedná [I don’t know what this is]*) if they did not know what the image was. All participants saw an image of a mouse as their first item, which was selected because it was very familiar according to Duñabeitia et al’s (2022) data, then rated a set of 100 items presented in randomised order.

### Data cleaning

We carried out a number of data quality checks to ensure that we detected any low-quality responses/participants; this process is visualised in Fig. 3. This was similar to the process used in Experiment 1, but we did not implement the calibrator and control item steps, as there was only one calibrator item and no controls (because control items for images was not possible).

1. *Image knowledge:* We checked for any participants who reported not knowing over 20% of the images they were presented with; however, there were no such participants.
2. *Straightlining:* If a participant straightlined on more than two of the four dimensions, we chose not to include them in the final dataset, resulting in the exclusion of 10 participants. Additionally, we removed a participant’s response to words on a specific dimension if they straightlined for over 95% of the images, resulting in the exclusion of 1,919 responses.
3. *Timing:* We inspected the time taken to complete the whole experiment by each participant, by log-transforming the durations. We again applied a filter of any participant who was quicker than the mean duration by 2.5 standard deviations, but there were no such participants. Once again, there was no upper cut-off point.

### Data summaries

We calculated the same summary statistics for each of the images for both colour and grayscale as those reported for Experiment 1. See Fig. 4 for a visualisation of the data and Table 1 for sample size summaries.

### Analysis and results

#### Reliability analysis

The internal consistency of the individual rating scales was assessed using Cronbach's alpha (Cronbach, 1951). We calculated alpha values for each list of images.^5^ The range of alpha values across the lists were as follows: gender = [.91,.95], location = [.88,.92], political = [.88,.93], valence = [.89,.95]. This indicates that the rating scales for the images can be considered to have high reliability, as was the case in Experiment 1.

#### Correlations between item versions

**Fig. 9:**
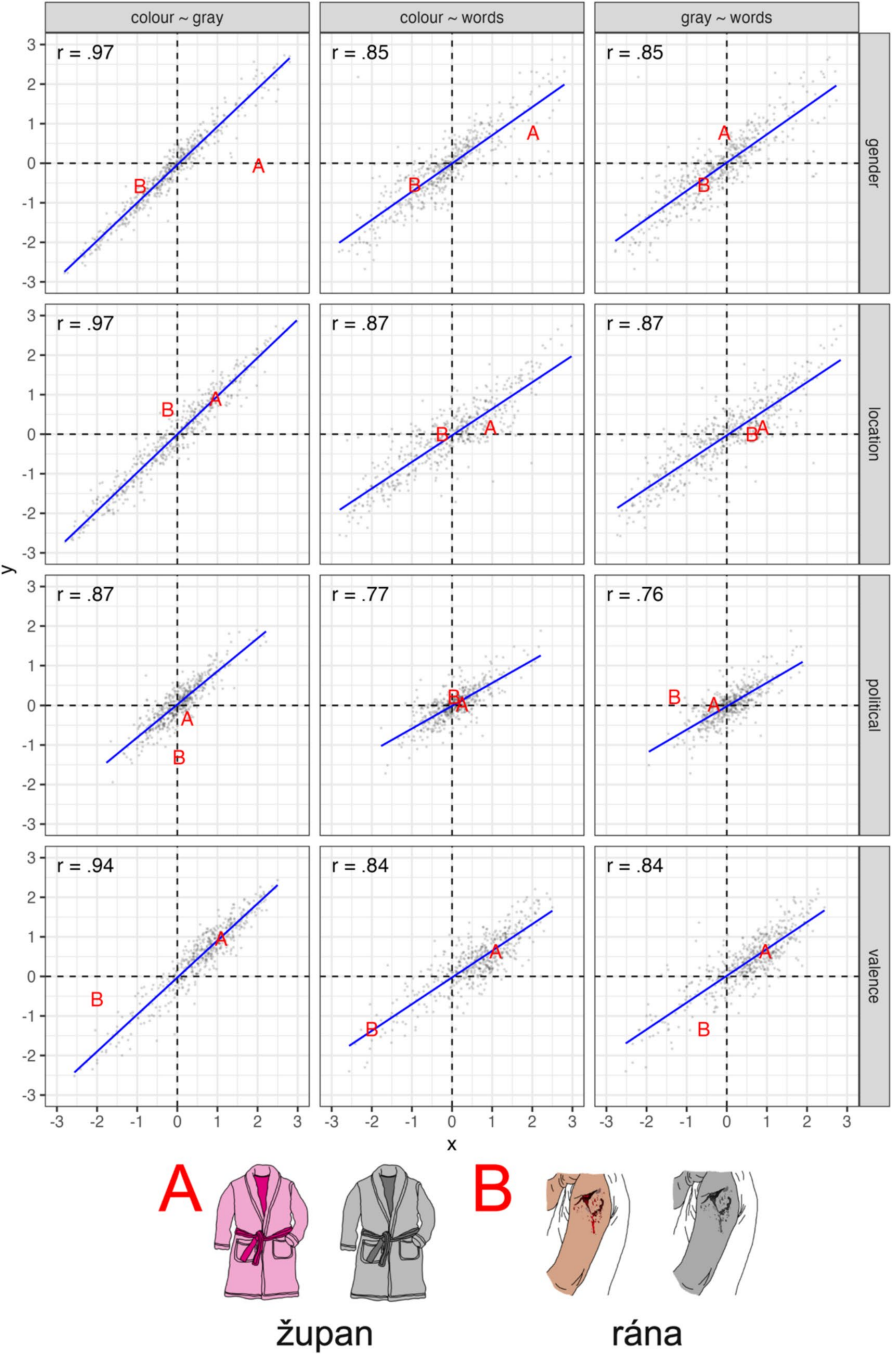
Comparisons between the colour, grayscale and word versions of the items from the Multilingual Picture Database (Duñabeitia et al., 2022). The facets on the top divide the data by stimuli comparison, with the mean rating values given on the x- and y-axis, respectively (e.g., colour ~ gray has ratings for items in colour on the x-axis and grayscale items on the y-axis). The facets on the right divide the data by the dimensions. The Pearson correlation coefficients are given in the top left corner of each facet, with a linear fit to the data added in blue. Two example stimuli are represented on the plots as red letters—A (dressing gown) and B (cut/wound)—with the colour, grayscale and Czech word versions shown at the bottom of the plot

In order to assess whether the socio-semantic norms differed across stimulus types (i.e. words/colour images/grayscale images), we inspected the correlations between the ratings for each of the dimensions. We analysed the data using Pearson’s correlation coefficients with each stimulus type comparison (i.e. colour ~ grayscale, colour ~ words, grayscale ~ words), with all *r*s >.76; see Fig. 9.

*Colour versus grayscale images*: The strongest correlations were found between the two image versions, with all *r*s >.87. Whilst this strong relationship might be expected given that stimuli only differ in terms of their visual appearance, there are items that exhibit notable variation. For example, in the gender dimension, the colour and grayscale versions of the image for a dressing gown have substantially different ratings, where the colour version displays the item in pink, and this has a more feminine association (*M* = 2.02), whereas the grayscale version is much more neutral (*M* = − 0.06); see item A in Fig. 9. This variation might be explained by the stereotyping of pink clothing being associated more with femininity, and when the colour bias is removed, the gender association bias might also be affected. However, there appear to be other sources of variation between colour and grayscale items; for example, the colour image of a cut (wound) (see item B in Fig. 9) has a much more negative rating for valence (*M* = − 2.00) than the grayscale version (*M* = − 0.58), but the grayscale version is much more liberal (*M* = − 1.31) than the colour version (*M* = 0.05) in the political dimension. This might be explained as a conceptual difference, as the grayscale image may be interpreted as a tattoo rather than a cut/wound, leading to two different semantic representations (note that the Duñabeitia et al. (2022) data were only normed based on the colour versions of the images).

*Images versus words*: The correlations between colour/grayscale images and words were slightly weaker than the colour ~ grayscale relationships, but were still interpreted as being very strong, indicating that—in general—images and words with the same conceptual meanings in our dataset capture very similar socio-semantic representations. However, there is again variation in the dataset, indicating that the alignment between ratings for words and images is not always stable. For example, *župan* is the Czech word for dressing gown and was rated as weakly associated with femininity (*M* = 0.78), whereas it was much more strongly associated with femininity in the colour version (*M* = 2.02), but close to neutral in the grayscale version (*M* = − 0.06). Moreover, the Czech word for cut/wound is *rána* and was rated very similarly to the colour version along all dimensions, but varied considerably from the grayscale version for valence and political dimensions, again suggesting that the grayscale version may have been interpreted as something conceptually different from the colour and word versions.

## General discussion

The SocioLex-CZ norms present a novel and innovative tool that can provide an important resource for a diverse range of scientific applications, such as understanding the role of these dimensions in semantic space, testing embodied theories of cognition, or considering which stimuli may be suitable for psycholinguistic and sociolinguistic experiments. The dataset is the largest known resource that captures conceptual associations along a multidimensional set of socially relevant dimensions of gender, location, political, valence and age, with a number of summary variables and the underlying raw data made freely available. Moreover, we provide ratings not only for a large set of words in Czech, which has typically received little coverage in terms of the norms currently available, but also for both colour and grayscale images, expanding the range of potential research applications that the norms can be used for. We hope that the dataset will create new synergies between the psychological, social and language sciences by opening up new research questions relating to socio-semantics, where our understanding of socially meaningful dimensions of semantic representations is explored and investigated in more detail.

Researchers have long been interested in how gender is represented semantically, with large-scale norms recently becoming available in English (Lewis et al., 2022; Scott et al., 2019) and Dutch (Vankrunkelsven et al., 2024). Our Czech norms add to this growing body of work, and as Czech is a grammatically gendered language, these norms may be of interest to researchers investigating whether grammatical and semantic gender interact, i.e. the linguistic relativity hypothesis (see Samuel et al., 2019). For example, Brand et al. (2025) have used the gender norms to explore whether explicit and implicit ways of measuring gender associations differ for different types of stimuli, specifically by investigating ratings made by Czech speakers in their L1 Czech, with Czech speakers in their L2 English, as well as with L1 English speakers.

Moreover, the large number of items in our norms may also facilitate further work investigating how abstract concepts are grounded in social aspects of meaning (Barsalou, 2020; Borghi et al., 2019; Pexman et al., 2023), with our location, political and age dimensions offering novel ways to quantify socio-semantic representations. For example, words like *demokracie* [democracy] and *riskantní* [risky] that are normally considered abstract have high association strength along these dimensions. Recent work that has investigated the concept of socialness has uncovered important insights into possible ways abstract concepts can be grounded through social experience (e.g. Diveica et al., 2023, 2024; Pexman et al., 2023). Although the variables presented in the current paper are not by definition the same as the broader definition used in socialness norms, they do encompass important features of socialness, such as social roles, characteristics, spaces and ideologies. These more specific dimensions of meaning could be used to further investigate the role of social semantics in the grounding of abstract concepts, and embodied theories of semantic representation more generally (Barsalou, 2020; Borghi et al., 2019).

We have demonstrated that the individual scales used to capture the socio-semantic dimensions are reliable, with strong agreement across raters, which is important for our new dimensions of location and political. Additionally, our analyses revealed a clear relationship between our word stimuli and existing norms for translating equivalent items in English for the dimensions of gender and valence, providing further support for their validity, as was the case in Scott et al. (2019) and Warriner et al. (2013). Our analyses of how the dimensions relate to each other has provided new empirical insights, revealing a number of interesting relationships that highlight how some concepts may co-vary in terms of their socio-semantic representations—i.e., concepts that are more liberal may also have associations with femininity, urban environments, positive emotions and younger age groups, whereas more conservative concepts may have associations with masculinity, rural environments, negative emotions and older age groups.

By providing data not only for words but also for colour and grayscale images, we have been able to assess the extent to which different versions representing the same underlying concept may vary in the way they represent socio-semantic meaning. Our analyses suggest generally strong alignment across the different stimuli versions, but we stress that not all items may be represented similarly, indicating that colour may bias the representation, or even lead to processing the item as something conceptually different. Thus, we emphasise the importance of considering how concepts may not align across different presentation modalities or versions, if only relying on norms derived from word labels.

Nevertheless, there are still a number of important directions for future research that will help improve the utility of socio-semantic norms. We acknowledge that our participant sample, although relatively large, is heavily skewed and restricted to a subset of the more general population. Specifically, we collected the data for our norms from a predominantly female sample of university students of the humanities, aged 18–30, with more liberal attitudes and urban affiliation. Although other norming studies have used a similar demographic sample (e.g. sensory modality norms by Speed and Brysbaert, 2024), the collection of norms where the dimensions relate to socially meaningful dimensions, such as gender, location, political and age, may be influenced by the socio-demographic profiles of the individual participants. The extent to which certain demographic groups (e.g. female vs male or young vs old) may differ remains a question requiring further attention. Although the primary focus of the current paper is to establish and introduce the first large-scale socio-semantic norms, we are currently collecting more data from more diverse samples of participants to address the question of whether socio-semantic representations are stable or vary across different socio-demographic groups.

Similarly, the extent to which the norms are predictive of measures that relate to linguistic or cognitive processing has only been initially assessed in this paper. Whilst there is evidence that some variables predict reaction times on a lexical decision experiment, these results would benefit from a more comprehensive analysis that takes into account different measures of processing, as well as exploring other linguistic/semantic effects, such as part of speech or concreteness. Emerging evidence suggests that this is the case for norms that quantify socialness (Diveica et al., 2023; Pexman et al., 2023). This would provide valuable additional empirical insights into whether social meaning acts as a significant contributor to the way concepts are organised, grounded and processed.

## Data Availability

The anonymised raw data and summary data, as well as the materials and stimuli from the experiments reported in this paper, can be accessed on the Open Science Framework at this link: 10.17605/OSF.IO/PV9MD

## Footnotes

1 To avoid ambiguity, we use the term location to refer only to the rural/urban distinction and not to spatial locations.

2 Simplified R syntax for latent mean models: clmm(rating_ordinal ~ 1 + (1|item) + (1|participant), data = dimension_ratings, link = "probit").

3 We excluded two words (*talmud* [Talmud] and *imám* [Imam]) from their respective lists, as these items were known by < 50% of the participants, resulting in missing values. We also excluded the control and calibrator words from this analysis, so that we only analysed the items that were assigned to the specific lists.

4 Note that the correlation here is negative, as the gender ratings of Scott et al. (2019) had masculine/feminine on opposite sides of the scale to our ratings.

5 We excluded five images (*PICTURE_146_gray, PICTURE_192_colour, PICTURE_192_gray, PICTURE_616_colour* and *PICTURE_616_gray*) from their respective lists, as these items were known by < 50% of the participants, resulting in missing values.

## Code availability

All code used in the data preprocessing, filtering and analysis from the experiments reported in this paper can be accessed on the Open Science Framework at this link: 10.17605/OSF.IO/PV9MD
